# Factors Affecting Stream Nutrient Loads: A Synthesis of Regional SPARROW Model Results for the Continental United States^1^

**DOI:** 10.1111/j.1752-1688.2011.00577.x

**Published:** 2011-10

**Authors:** Stephen D Preston, Richard B Alexander, Gregory E Schwarz, Charles G Crawford

**Keywords:** computational methods, geospatial analysis, statistical models, nutrients, nonpoint-source pollution, point-source pollution, watershed management, watersheds, rivers/streams, landscape, modeling, empirical modeling, SPARROW models

## Abstract

**Abstract:**

We compared the results of 12 recently calibrated regional SPARROW (SPAtially Referenced Regressions On Watershed attributes) models covering most of the continental United States to evaluate the consistency and regional differences in factors affecting stream nutrient loads. The models – 6 for total nitrogen and 6 for total phosphorus – all provide similar levels of prediction accuracy, but those for major river basins in the eastern half of the country were somewhat more accurate. The models simulate long-term mean annual stream nutrient loads as a function of a wide range of known sources and climatic (precipitation, temperature), landscape (e.g., soils, geology), and aquatic factors affecting nutrient fate and transport. The results confirm the dominant effects of urban and agricultural sources on stream nutrient loads nationally and regionally, but reveal considerable spatial variability in the specific types of sources that control water quality. These include regional differences in the relative importance of different types of urban (municipal and industrial point *vs.* diffuse urban runoff) and agriculture (crop cultivation *vs.* animal waste) sources, as well as the effects of atmospheric deposition, mining, and background (e.g., soil phosphorus) sources on stream nutrients. Overall, we found that the SPARROW model results provide a consistent set of information for identifying the major sources and environmental factors affecting nutrient fate and transport in United States watersheds at regional and subregional scales.

## Introduction

Excessive nutrient loading is well established as the primary cause of eutrophication of coastal estuaries as well as freshwater streams and lakes (NRC, 2000). A variety of nitrogen and phosphorus sources including upstream urban areas, sewage treatment plants, and agricultural land all contribute to stream loads of these nutrients which can affect water quality in receiving waters such as coastal estuaries. Management of water quality in receiving waters is complicated by the fact that the sources of nutrients vary by type, magnitude, and location, and are distributed over large areas and across multiple jurisdictions. Further complications result from spatial gaps in stream monitoring, for which data typically are collected in too few locations to adequately support state and local management decisions (GAO, 2000). Mathematical models provide a means of interpreting and extrapolating monitoring data to mitigate limitations in the quantity of available data and to improve understanding of the environmental factors that affect water quality over large spatial scales and diverse geographic settings. The United States Geological Survey's (USGS) SPAtially Referenced Regressions On Watershed attributes (SPARROW) model was developed to aid in the interpretation of monitoring data and simulate water-quality conditions in streams across large spatial scales (Smith *et al.*, 1997). SPARROW has been used previously to assess stream nutrient loading for the continental United States (U.S.) (Smith *et al.*, 1997; Smith and Alexander, 2000), regionally for large watersheds in the U.S. (e.g., Preston and Brakebill, 1999; Alexander *et al.*, 2000, 2008), and internationally (Alexander *et al.*, 2002). New regional-scale SPARROW nutrient models for the conterminous U.S. have been recently developed and are highlighted in the Featured Collection of papers in this issue of the *Journal of the American Water Resources Association* (Preston *et al.*, 2009; Preston *et al.*, this issue).

A key question in developing a SPARROW model or any other water-quality model over large watershed scales is whether the governing material transport equations should differ spatially in their functional forms and/or coefficient values to account for the effects of heterogeneity and scale. Despite advances in watershed science to address this question, uncertainties remain over the nature of the effects of spatial heterogeneities in landscape properties and processes at all scales on material transport and associated model descriptions (e.g., McDonnell *et al.*, 2007; Kirchner, 2009; Rode *et al.*, 2010). Limitations in the availability, consistency, and accuracy of geospatial data over large areas also restrict the ability to adequately understand and simulate water-quality processes at such scales.

For SPARROW, the complexity of the transport functions and variables is governed by the environmental conditions in the geographic domain of the model, the availability and resolution of the geospatial data for describing these conditions, and the intended uses of the model for research and management. SPARROW models are frequently applied over large geographic areas with unchanging parameter values because the parameters can be reliably estimated and interpreted, and can be generally applied to a broad range of environmental settings. Such models have the advantage of including large numbers of calibration sites and covering a broad gradient in the environmental properties that affect water quality, features that enhance both the quantity and quality of the data for statistically estimating and applying the model. However, models with greater geographic specificity can potentially provide improved accuracy and utility for understanding processes and supporting water-resource management for selected watersheds and environmental conditions over smaller spatial scales. For example, Schwarz *et al.* (this issue) demonstrates potential improvements in the accuracy of previous national SPARROW models through the use of regional coefficients, thus demonstrating the potential for constructing models with greater regional specificity.

Here, we present a synthesis of the calibration and simulation results from 12 independently calibrated regional-scale SPARROW models that describe water-quality conditions throughout major drainage basins of the conterminous U.S. We find that the results of our analysis of the regional model predictions are generally consistent with our expectations that the predominant sources and processes controlling nutrient loads in streams display many similarities across broad regions of the U.S. However, the analysis reveals new insights about important geographical differences in these controls and their effects on the response of stream nutrient loads as described by regional differences in the model coefficients and predictions of nutrient yields and sources.

Our assessment evaluates regional SPARROW nutrient models recently developed as part of a nationwide investigation of stream nutrients sponsored by the USGS National Water Quality Assessment (NAWQA) Program and reported in this Featured Collection. These models are intended to support management needs in six major regions of the conterminous U.S. using regionally specific information describing water-quality conditions. The models provide an opportunity to evaluate the evidence for regional variability in the environmental factors and processes that affect stream nutrient loads. Specifically, our analysis identifies the environmental characteristics that affect nutrient levels in streams nationally and regionally, and evaluates the consistency in the predicted spatial patterns of sources of nutrients to streams and losses of nutrients in terrestrial and aquatic ecosystems. Our analysis is intended to provide a continental and regional-scale perspective on current questions in watershed science about how model structure and complexity are affected by spatial heterogeneity and scale. However, because the analysis is a postcalibration evaluation, some of the major similarities and differences in the regional models can be only qualitatively evaluated. This approach is complementary to that of Schwarz *et al.* (this issue), which quantitatively evaluates evidence for regional specificity using a previously published national SPARROW model based on 1992 nutrient sources (Alexander *et al.*, 2008). By contrast, we present a meta-analysis of independently estimated regional models, based on the use of more recent geospatial data (e.g., 2002 sources) and a more extensive collection of local, state, and federal stream monitoring records from across the conterminous U.S. Collectively, these new geospatial data provide much more detailed descriptions of landscape and water-quality conditions to assess regional variability in model specifications and interpretability.

## Methods

SPARROW models are designed to provide information that describes the spatial distribution of water quality and that of related environmental characteristics. Models are developed by statistically relating measured stream nutrient loads with geographic characteristics observed in the watershed (see Schwarz *et al.*, 2006 for a detailed presentation of the SPARROW model structure and methods). Data describing geographic characteristics are obtained from spatially detailed national databases because they tend to be systematically compiled over large regional scales. Information on nutrient loads, however, is usually developed from locally collected water-quality and discharge data, and is specific to the modeled region (Preston *et al.*, 2009). The overall structure of SPARROW models is based on fundamental hydrological and biogeochemical principles, but the final formulation and complexity of each model are determined by a statistical calibration process and by evaluations of model performance and interpretability. Many watershed variables are selected from a larger set of potential explanatory variables and assessed for their utility in explaining the spatial variability in measured stream load. Only those variables that are statistically significant and physically interpretable are retained and represented in the models. Thus, SPARROW models serve as statistical tools for identifying the environmental conditions and landscape properties that are important controls on stream nutrient loads within the model's geographic domain.

**FIGURE 1:**
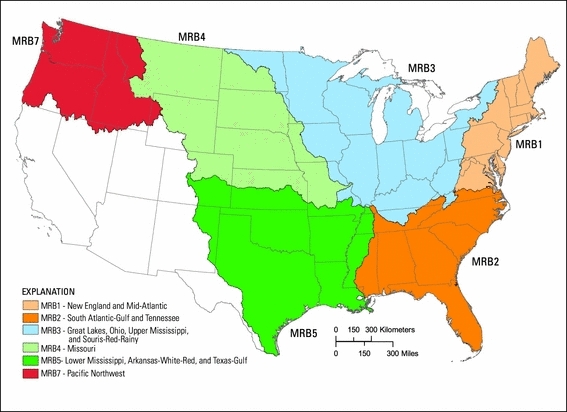
USGS NAWQA Regions for SPARROW Nutrient Model Development.

The process for developing SPARROW models is designed to provide unique identification of the factors affecting water quality and their relative importance through the combined use of a mechanistic model structure and statistical estimation of all model coefficients. This is accomplished by: (1) imposing process constraints such as mass balance, first-order nonconservative transport, and the use of digital topography and hydrologic networks that provide spatially explicit descriptions of water flow paths; (2) using observed data (i.e., long-term measurements of streamflow, water quality, and geospatial data on watershed properties) to inform the complexity of the model so that only statistically significant explanatory variables, which are uncorrelated with one another, are selected; (3) imposing physically based constraints on source coefficients, which are limited to be positive; and (4) utilizing various statistical diagnostics to detect the potential for nonunique solutions and misspecification of the models. The models are also evaluated for accuracy through comparisons of the model predictions of stream nutrient yields and estimates of the rates of nutrient removal in streams and reservoirs with available literature estimates. We believe these approaches provide model results that are much more defensible in their uniqueness than those typically found for mechanistic simulation models and more interpretable than those associated with conventional linear regression models. Our assessment of regional variability in the factors affecting water quality is based on the results of simulations made with recently calibrated SPARROW nutrient models that were developed for large regions of the country designated as “major river basins” (MRBs) (Figure 1). Models were developed for seven of the eight major river basins that cover the spatial extent of the continental U.S. As of the date of this article, however, nutrient models have been developed for only six of the eight basins; salinity was the focus of modeling in the arid southwest (MRB6), and no models have been developed to date for California (MRB8). Thus, total nitrogen (TN) and total phosphorus (TP) models were developed for 6 large regions, giving a total of 12 models that cover most of the continental U.S. These models provide predictions of long-term mean annual water-quality conditions and nutrient sources that are specific to each region, but through model comparisons can also be used to evaluate factors that affect water quality broadly across regions.

All of the regional models are similar in structure, functional relationships and types of geospatial data, and are similar in the same aspects to previously developed national SPARROW models (e.g., Alexander *et al.*, 2008). All contain three major explanatory components, including nutrient sources expressed as mass input per unit time or the area of specific land uses, exponential land-to-water delivery functions, and first-order aquatic decay relationships. With the exception of one of the six regions (the Northeast – MRB1), the models are based on similar river network data, which are linked to digital topography for the watersheds. The models were calibrated using available stream loading data that were collected by federal, state, and local agencies, and which reflect conditions within the regions (Saad *et al.*, this issue). In most cases, explanatory variable data were defined using nationally consistent geospatial datasets (Wieczorek and Lamotte, 2011a,b;). However, in a few cases explanatory variable data were developed from geospatial datasets that are unique to the modeled region. One example is the dataset that describes the spatial distribution of Pacific Northwest alder trees which are known to fix significant amounts of nitrogen (Wise and Johnson, this issue). In general though, each of the models is based on region-specific stream load estimates used for model calibration and explanatory variable information from nationally consistent geospatial datasets. Thus, variability in the factors affecting nutrient loads among regions is potentially reflected by differences in the explanatory variables identified as statistically significant predictors in the three source-transport structural components, by the estimated values of the parameters associated with the variables, and by the resulting model predictions of nutrient contributions to streams (mass per unit area, source shares) and removal in terrestrial and aquatic ecosystems. We highlight differences in the model simulation results that are caused by both regional differences in environmental conditions and differences in the parameters identified statistically as part of the model calibration process.

To evaluate the evidence for model consistency nationally or differences across multiple regions, the model simulation results are compiled in several ways to allow informed comparisons to be made.

1. Calibration diagnostics are compared to assess consistency in the fit of the models and whether differences in fit might potentially confound our ability to make reliable comparisons of the model predictions.
2. To more thoroughly evaluate differences in the accuracy of the regional models (i.e., ability of the models to describe spatial variability in measured stream load), we perform a regression analysis of the combined set of SPARROW model residuals (see Supporting Information for details). In that analysis, the SPARROW model residuals are the dependent variables and regional binary terms are the explanatory variables that are used to detect statistically significant differences among regions.
3. Model calibration results are evaluated for consistency and regional specificity in the sources that affect stream nutrient loads. We accomplish this (evaluation) by comparing the types of sources identified as statistically significant (α = 0.05) in the models. We then evaluate the magnitude of the predicted source contributions through the use of maps and tabular information that illustrate the spatial differences and similarities in the factors affecting nutrient loads.
4. We evaluate the regional models for consistency in the variables that enhance or attenuate nutrient delivery to streams by affecting flow paths (runoff, groundwater) or rates of transport. Differences in the magnitude of landscape delivery rates among the regional models are evaluated by mapping the spatial distribution of aggregate model delivery rates that account for the effects of all landscape delivery factors. The aggregate delivery is simply defined as the amount of a nutrient exported from an individual drainage divided by the amount of that nutrient input to that drainage. To ensure consistency across all models, the ratio for the TN models is reported only for agriculture and atmospheric deposition, and the ratio for the TP models is reported only for agriculture. These source terms were used because they are the only ones that are both affected by landscape delivery and common to all the models. For this analysis, they are used primarily to estimate landscape delivery rates and not necessarily to draw inferences about these particular sources.
5. We compare the magnitudes of the estimates of the mean rates of instream and reservoir nutrient loss quantified by the different regional models; a consistent first-order aquatic decay expression was used as part of all models.

## Results

We present information on the performance of the regional models (Table 1) and their parameters in a series of separate tables for each of the source-transport structural components (Tables 2-5). Details of the calibration procedures for each model can be found in the individual papers in this issue (see Table 1 for references to the papers describing each model). Except where noted, the selection of parameters included in each of the regional models was made on the basis of a statistical significance level of 0.05. The results are organized in sections that correspond with the primary structural components of the models.

**TABLE 1:** Comparison of Regional SPARROW Model Fit Statistics

		MRB1	MRB2	MRB3	MRB4	MRB5	MRB7
							
Nutrient	Fit Statistic	Moore *et al.* (this issue)	Hoos and McMahon (2009) (TN); García *et al.* (this issue) (TP)	Robertson and Saad (this issue)	Brown *et al.* (this issue)	Rebich *et al.* (this issue)	Wise and Johnson (this issue)
Total nitrogen	Number of calibration sites (*n*)	363	321	708	193	344	178
	Average area per site (km^2^/site)	1,223	2,559	2,032	6,856	4,025	3,787
	Root mean square error (RMSE)	0.345	0.320	0.408	0.744	0.552	0.640
	Coefficient of determination (*R*^2^) of load estimate	0.97	0.96	0.95	0.90	0.92	0.89
	Coefficient of determination (*R*^2^) of yield estimate	0.83	0.72	0.85	0.84	0.86	0.76
Total phosphorus	Number of calibration sites (*n*)	457	370	810	311	442	228
	Average area per site (km^2^/site)	971	2,208	1,695	4,254	3,167	3,150
	Root mean square error (RMSE)	0.651	0.539	0.493	1.010	0.743	0.693
	Coefficient of determination (*R*^2^) of load estimate	0.91	0.91	0.93	0.84	0.88	0.86
	Coefficient of determination (*R*^2^) of yield estimate	0.60	0.67	0.73	0.68	0.80	0.71

Note: MRB, major river basin; TN, total nitrogen; TP, total phosphorus.

**TABLE 2:** Comparison of Total Nitrogen (A) and Total Phosphorus (B) Sources Identified by Regional SPARROW Models

A. Total Nitrogen^1^									

Nitrogen Source Category	Predictor Variable Description	Coefficient Units	Statistical Measures	MRB1	MRB2	MRB3	MRB4	MRB5	MRB7
Point source	Permitted wastewater discharge (kg/year)	Dimensionless	Estimate	1.156	0.786	0.789	0.962	1.390	1.597
			Standard error	0.175	0.090	0.113	0.328	0.271	0.822
			Significance level	<0.001	<0.001	<0.001	0.002	<0.001	0.027
Urban land	Area of impervious surfaces (km^2^)	kg/km^2^/year	Estimate		2,470				
			Standard error		649				
			Significance level		<0.001				
	Area of developed land (km^2^)	kg/km^2^/year	Estimate	1,422			511	609	941
			Standard error	169			256	152	270
			Significance level	<0.001			0.024	<0.001	<0.001
Agricultural fertilizer	Commercial fertilizer applied to agricultural land (kg/year)	Dimensionless	Estimate		0.110	0.131	0.036	0.061	0.048
			Standard error		0.020	0.038	0.014	0.013	0.020
			Significance level		<0.001	<0.001	0.005	<0.001	0.008
	Commercial fertilizer applied to corn/soybeans/alfalfa (kg/year)	Dimensionless	Estimate	0.310					
			Standard error	0.039					
			Significance level	<0.001					
	Commercial fertilizer applied to “other” crops (kg/year)	Dimensionless	Estimate	0.186					
			Standard error	0.081					
			Significance level	0.011					
Agricultural livestock	Manure from livestock production (kg/year)	Dimensionless	Estimate	0.090	0.050		0.040		0.113
			Standard error	0.026	0.020		0.019		0.048
			Significance level	<0.001	0.010		0.018		0.010
	Manure from confined livestock production (kg/year)	Dimensionless	Estimate			0.291		0.169	
			Standard error			0.055		0.055	
			Significance level			<0.001		0.001	
	Manure from unconfined livestock production (kg/year)	Dimensionless	Estimate					0.075	
			Standard error					0.028	
			Significance level					0.004	
Other agricultural sources	Area of catchment with agricultural land use (km^2^)	kg/km^2^/year	Estimate			625			
			Standard error			297			
			Significance level			0.018			
Atmospheric deposition	Wet deposition of inorganic nitrogen (kg/year)	Dimensionless	Estimate	0.279	0.500	0.513	0.040	0.216	0.099
			Standard error	0.028	0.050	0.040	0.025	0.041	0.078
			Significance level	<0.001	<0.001	<0.001	0.057	<0.001	0.105
Forest sources	Area of forest land west of the Cascade mountain range (km^2^)	kg/km^2^/year	Estimate						78.3
			Standard error						30.8
			Significance level						0.006
	Area of forest land east of the Cascade mountain range (km^2^)	kg/km^2^/year	Estimate						115
			Standard error						25
			Significance level						<0.001
	Nitrogen fixation by alder forests (tree basal area – m^2^)	kg/m^2^/year	Estimate						0.311
			Standard error						0.199
			Significance level						0.060

B. Total Phosphorus^1^									

Phosphorus Source Category	Predictor Variable Description	Coefficient Units	Statistical Measures	MRB1	MRB2	MRB3	MRB4	MRB5	MRB7
Point source	Permitted wastewater discharge (kg/year)	Dimensionless	Estimate	1.317	0.672	1.068	0.860	1.856	1.034
			Standard error	0.224	0.124	0.142	0.327	0.407	0.498
			Significance level	<0.001	<0.001	<0.001	0.004	<0.001	0.020
Urban land	Area of developed land (km^2^)	kg/km^2^/year	Estimate	106.3	88.0	52.3	32.3	106.1	61.7
			Standard error	14.2	17.4	14.4	13.1	22.8	23.6
			Significance level	<0.001	<0.001	<0.001	0.007	<0.001	0.005
Agricultural land	Area of catchment with agricultural land use (km^2^)	kg/km^2^/year	Estimate		48.4				
			Standard error		13.8				
			Significance level		<0.001				
Agricultural fertilizer	Commercial fertilizer applied to agricultural land (kg/year)	Dimensionless	Estimate			0.029	0.011	0.058	
			Standard error			0.004	0.005	0.013	
			Significance level			<0.001	0.027	<0.001	
	Commercial fertilizer applied to corn/soybeans/alfalfa (kg/year)	Dimensionless	Estimate	0.070					
			Standard error	0.019					
			Significance level	<0.001					
	Commercial fertilizer applied to “other” crops (kg/year)	Dimensionless	Estimate	0.230					
			Standard error	0.083					
			Significance level	0.003					
Agricultural livestock	Manure from livestock production (kg/year)	Dimensionless	Estimate	0.056	0.013		0.009	0.019	
			Standard error	0.010	0.005		0.004	0.005	
			Significance level	<0.001	0.003		0.006	<0.001	
	Manure from confined livestock production (kg/year)	Dimensionless	Estimate			0.086			
			Standard error			0.011			
			Significance level			<0.001			
	Manure from unconfined livestock production (kg/year)	Dimensionless	Estimate			0.032			
			Standard error			0.010			
			Significance level			<0.001			
Other agricultural sources	Fertilizer and manure applied to agricultural land (kg/year)	Dimensionless	Estimate						0.018
			Standard error						0.008
			Significance level						0.012
Mining	Area of catchment with mining land use (km^2^)	kg/km^2^/year	Estimate		0.333				
			Standard error		0.119				
			Significance level		0.003				
Background sources	Geologic sources as indicated by stream bed TP levels (dimensionless)	Dimensionless	Estimate		0.037				0.015
			Standard error		0.008				0.002
			Significance level		<0.001				<0.001
	Generated from channel erosion, m^3^/s (length – km)	km^−1^	Estimate				0.176	0.034	
			Standard error				0.034	0.014	
			Significance level				<0.001	0.008	
	Area of catchment covered by forested land (km^2^)	kg/km^2^/year	Estimate	11.4		14.7		2.04	
			Standard error	1.7		1.72		1.26	
			Significance level	<0.001		<0.001		0.050	

Notes: Values represent the coefficient estimates for the models, their standard error, and their significance levels. MRB, major river basin; TP, total phosphorus.

1 Most explanatory variables are defined/derived consistently for each of the regional models. However, for a few the definitions/derivations differ slightly among the regional models. Please see the individual model papers (references listed in Table 1) for the specific details of how the explanatory variable data were developed for each model.

**TABLE 3:** Landscape Characteristics Identified by Regional SPARROW Models as Statistically Significant Total Nitrogen (A) and Total Phosphorus (B) Delivery Variables

Type of Delivery Effect	MRB1	MRB2	MRB3	MRB4	MRB5	MRB7

A. Total nitrogen^1^						
Landscape characteristics associated with enhanced delivery to streams	ln (ratio of nitrate to ammonium in nitrogen deposition)	Mean annual precipitation (mm)	Mean annual precipitation (mm)	Mean annual precipitation (mm)	Mean annual precipitation (mm)	Effective mean annual precipitation (mm)
	Northern Piedmont ecoregion indicator (0,1)	Percentage of catchment in hydrologic region 4 (%)	Stream drainage density (km/km^2^)	Percentage of drainage area designated as having loess geology (%)	Infiltration excess overland flow (%)	
	Valley and ridge physiographic province indicator (0,1)	Percentage of catchment in hydrologic regions 6, 9, or 11 (%)	Percentage of drainage area with tile drains (%)			
			Soil clay percentage (%)			
Landscape characteristics associated with reduced delivery to streams	Mean annual temperature (^o^C)	Depth to bedrock (cm)	Mean annual temperature (^o^C)	Mean annual temperature (^o^C)		Percentage of catchment in hydrologic region 20 (%)
	Average overland flow distance to the stream channel (km)	Percentage of catchment in hydrologic region 2 (%)		Percentage of drainage area used as irrigated agricultural land (%)		
		Percentage of catchment in hydrologic region 7 (%)				
		Percentage of catchment in hydrologic region 16 (%)				

B. Total phosphorus^1^						
Landscape characteristics associated with enhanced delivery to streams	Eastern Great Lakes and Hudson Lowlands ecoregion indicator (0,1)	Mean annual precipitation (mm)		Mean annual precipitation (mm)	Mean annual precipitation (mm)	Effective mean annual precipitation (mm)
		Soil K factor		Average slope in catchment (%)	Infiltration excess overland flow (%)	
		Soil pH			Soil K factor	
Landscape characteristics associated with reduced delivery to streams	Percentage of streamflow coming from groundwater (%)	Water table depth (m)	Soil permeability (cm)	Soil permeability (cm)		Soil permeability (cm)
	Average overland flow distance to the stream channel (km)	Soil organic matter	Percentage of drainage area with tile drains (%)			Percentage of catchment in hydrologic region 20 (%)
	New England coastal zone physiographic province indicator (0,1)					

Note: MRB, major river basin.

1 Most explanatory variables are defined/derived consistently for each of the regional models. However, for a few the definitions/derivations differ slightly among the regional models. Please see the individual model papers (references listed in Table 1) for the specific details of how the explanatory variable data were developed for each model.

**TABLE 4:** Comparison of Regional SPARROW Model Instream-Loss Coefficient Estimates

Predictor Variable Type	Predictor Variable Description	Coefficient Units	Statistical Measures	MRB1	MRB2	MRB3	MRB4	MRB5	MRB7
Total nitrogen instream attenuation rate estimates	Time of travel in each stream reach where mean discharge <1.13 m^3^/s (days)	(days)^−1^	Estimate			0.424			
			Standard error			0.100			
			Significance level			<0.001			
	Time of travel in each stream reach where mean discharge <1.42 m^3^/s (days)	(days)^−1^	Estimate					0.365	
			Standard error					0.082	
			Significance level					<0.001	
	Time of travel in each stream reach where mean discharge <2.83 m^3^/s (days)	(days)^−1^	Estimate	0.224					
			Standard error	0.144					
			Significance level	0.060					
	Time of travel in each stream reach where mean discharge <3.11 m^3^/s (days)	(days)^−1^	Estimate				0.150		
			Standard error				0.057		
			Significance level				0.004		
	Time of travel in each stream reach where mean discharge >1.13 and <1.98 m^3^/s (days)	(days)^−1^	Estimate			0.233			
			Standard error			0.096			
			Significance level			0.016			
	Time of travel in each stream reach where mean discharge >1.42 and <28 m^3^/s (days)	(days)^−1^	Estimate					0.079	
			Standard error					0.021	
			Significance level					<0.001	
	Time of travel in each stream reach where mean discharge <28 m^3^/s (days)	(days)^−1^	Estimate		0.140				
			Standard error		0.050				
			Significance level		<0.001				
	Time of travel in each stream reach where mean discharge >28 m^3^/s (days)	(days)^−1^	Estimate		0.014				
			Standard error		0.020				
			Significance level		0.260				
Total phosphorus instream attenuation rate estimates	Time of travel in each stream reach where mean discharge <1.42 m^3^/s (days)	(days)^−1^	Estimate			0.198		0.254	
			Standard error			0.072		0.067	
			Significance level			0.006		<0.001	
	Time of travel in each stream reach where mean discharge >1.42 and <2.27 m^3^/s (days)	(days)^−1^	Estimate			0.298			
			Standard error			0.100			
			Significance level			0.003			
	Time of travel in each stream reach where mean discharge <13.4 m^3^/s (days)	(days)^−1^	Estimate						0.093
			Standard error						0.052
			Significance level						0.038
	Time of travel in each stream reach per meter of stream depth (days/m)	(days/m)^−1^	Estimate		0.048				
			Standard error		0.028				
			Significance level		0.085				

Notes: Values represent the coefficient estimates for the models, their standard error, and their significance levels. MRB, major river basin.

**TABLE 5:** Comparison of Regional SPARROW Model Reservoir Loss Coefficient Estimates

Predictor Variable Type	Predictor Variable Description	Coefficient Units	Statistical Measures	MRB1	MRB2	MRB3	MRB4	MRB5	MRB7
Total nitrogen	Reservoir loss; inverse hydraulic load (m/year)	(m/year)^−1^	Estimate		10.7	6.7	10.5	12.1	
			Standard error		2.20	1.45	4.36	2.75	
			Significance level		<0.001	<0.001	0.008	<0.001	
Total phosphorus	Reservoir loss; inverse hydraulic load (m/year)	(m/year)^−1^	Estimate	2.7	29.8	4.8	39.3	8.7	
			Standard error	2.40	8.44	1.12	12.49	2.62	
			Significance level	0.132	<0.001	<0.001	0.001	0.001	

Notes: Values represent the coefficient estimates for the models, their standard error, and their significance levels. MRB, major river basin.

### Model Calibration

A variety of measures of model performance are presented in Table 1. For the purpose of this discussion, we define “quality of fit” of the models in terms of well-known model fit statistics. Fit can be evaluated, in part, by quantifying the root mean square error (RMSE), which is simply the square root of the mean squared difference between the log-transformed measured and predicted values. Fit can also be assessed by evaluating the coefficient of determination (*R*^2^) for both load and yield predictions. The latter metric removes the influence of basin size on the *R*^2^ value and provides a more size-independent metric for assessing the ability of the model to account for the spatial variability in water quality. Table 1 also includes information on the number of sites used for calibration of each model and a measure of the density of sites representing each model region. We define site density in terms of average area (in km^2^) per site or the area represented by each site if all were distributed equally in space throughout the model region.

In general, the fit statistics indicate similar prediction accuracy among the models, but the differences in quality may be related to the number of sites available for calibration (Table 1). In all cases, the load *R*^2^ values for both TN and TP models are ≥0.84, and 8 of the 12 models have load *R*^2^ values that are ≥0.90, indicating that the models account for most of the spatial variability in log-transformed stream load in all model regions. As expected, the *R*^2^ values for yield are lower, and range from 0.60 to 0.86. This indicates that the models still account for most of the spatial variability in water quality even after accounting for the relationship of basin size to stream load. Error is higher in the TP models, presumably due to the greater temporal and spatial variability commonly observed in measurements of TP (than of TN) and to the greater complexity of transport processes involving sediment-adsorbed phosphorus. Error is also greatest among those models with fewest sites available for calibration. More precisely, those models with greater average area per site tend to have higher RMSE values (e.g., MRB4) and those with lower average area per site tend to have lower RMSE values (e.g., MRB2).

Results of the regression analysis of the combined set of residuals (i.e., prediction errors) from the regional SPARROW models indicate that, in most cases, the “regional effect” variables in the regression model are statistically significant predictors of the magnitude of the SPARROW prediction errors; this indicates that the magnitude of the prediction error in the SPARROW models varies significantly by region (see Table S1 in Supporting Information for details). For example, the average prediction error of the regional models, expressed as a percent of the mean nutrient load (based on one standard deviation of variation), ranges from 21 to 69% for the TN models and from 46 to 96% for the TP models. There is also a prominent east/west pattern in the magnitude of the prediction error of the models. Models for the easternmost regions (MRBs 1, 2, and 3) have a *negative*“regional effect” coefficient, implying that the SPARROW model prediction error is lower than the average error. By contrast, models for the western basins (MRBs 4, 5, and 7) in almost all cases have *positive*“regional effect” coefficients, implying higher than average SPARROW prediction error and lower prediction accuracy. Processes related to nutrient (and sediment) transport in the arid West may be more temporally and spatially variable than those in other regions, and the density of monitoring sites in the western states tends to be more sparse than in eastern parts of the U.S. (Table 1); the combination of these two factors may be the cause of greater uncertainty in the SPARROW models for the western regions. Nevertheless, the spatial consistency of these findings for both the TN and TP models suggests that the true unobserved processes that cause these differences are likely to be the same for both nutrient species.

### Model Identification of Nutrient Sources

The calibration results for the source variables are summarized for each regional model in Table 2, which includes the statistically significant source variables for the models along with their parameter estimates, standard errors, and significance levels. Each model parameter quantifies the marginal or incremental change in stream nutrient load in response to a unit change in the explanatory variable. For example, the coefficient associated with fertilizer use quantifies the magnitude of the change in stream load, in kilograms per year delivered to a stream (i.e., the load associated with the “incremental” drainage area of the reach), that occurs in response to a kilogram per year change in fertilizer use. Several categories of nutrient source variables are consistently found to be statistically significant in explaining the spatial variability in stream nutrient loads (Table 2), including effluent from point sources, and sources associated with developed land and agriculture.

In most of the models, point-source parameter estimates were both highly significant in accounting for the spatial pattern in water quality and close to expected values based on the functional form of the model. In most cases, the statistical significance levels (i.e., “*p*-values”) for point sources were ≤0.004, which indicates the strong statistical relationship between estimates of point-source nutrient contributions and downstream loads. Theoretically, point-source coefficient estimates should approximate the value 1, because point sources (quantified as mass per time) discharge directly to streams without the potential for land-based attenuation (Schwarz *et al.*, 2006). The regional model point-source coefficient estimates range approximately from 0.67 to 1.9, with only 3 of the 12 models (1 TN and 2 TP) indicating a mean value that is statistically different from the expected value of 1 (based on a two-tailed *t*-test for α = 0.05). Point-source coefficient estimates that are higher or lower than expected likely result from inaccurately estimated point-source contributions, which are empirically adjusted by the model to best match the observed stream loads. The available point-source data derived from the U.S. Environmental Protection Agency (USEPA) Permit Compliance System (PCS) database must often be estimated due to lack of reporting, thus potentially causing lower accuracy and precision in the SPARROW prediction of point-source contributions to the stream nutrient load (Maupin and Ivahnenko, this issue). Models with coefficient values <1 may also reflect the presence of large point sources discharging to streams that are smaller than those described by the resolution of the hydrologic network. In these cases, there is potentially instream attenuation that is not accounted for in the model.

One exception to the pattern described above is observed in the results of modeling in the Pacific Northwest (MRB7), where point-source estimates were statistically less significant explanatory variables (*p*= 0.027 for TN and *p*= 0.020 for TP) than in the other models. This may be due to the fact that most of the Pacific Northwest is sparsely populated and most point sources of nutrients are far downstream of monitoring sites, near where streams discharge to tidal waters (Wise and Johnson, this issue). Generally, SPARROW relates upstream effects to measured loads and may not capture the effects of sources that are not located above monitoring sites. A second possible explanation for the statistically weaker point-source terms in the model is that the accuracy of point-source data may not be adequate to fully characterize their effect in the Pacific Northwest region (Maupin and Ivahnenko, this issue).

Definitions of developed land as a measure of diffuse urban sources differed among the models, but in nearly all cases these measures, including impervious area (a more refined measure of the surficial properties of developed land), were statistically significant predictors of stream nutrient load (Table 2 shows five of six models for TN and all six for TP). Developed land serves as a surrogate measure of various diffuse urban sources in the model potentially including nutrient runoff from impervious surfaces and inflows from groundwater in urbanized catchments related to the use of fertilizers, septic systems, and atmospheric deposition from vehicle emissions. For TN models, the coefficient estimates range from 511 to 2,470 kg/km^2^/year. Impervious area was highly significant (<0.001) in the MRB2 model where the coefficient estimate was 2,470 kg/km^2^/year. The coefficient estimate for impervious area might be expected to be higher than for developed land because it accounts only for that portion of developed land that generates most of the urban runoff. For TP, the developed land coefficient estimates range from 32.3 to 106.3 kg/km^2^/year. These model estimates generally fall within the literature ranges reported for nitrogen and phosphorus for small urban dominated catchments (Beaulac and Reckhow, 1982; NRC, 2009).

Agricultural variables were determined to be significant predictors of stream nutrient loads in all of the regional models, but more refined measures such as crop-based fertilizer use were found to be better predictors in some of the models (Table 2). Estimates of fertilizer use are derived from fertilizer sales and crop distribution databases and represent the intensity of nutrient inputs to agricultural crops. In addition to accounting for actual fertilizer use, they may also serve as a proxy for other nutrient inputs and the effects of farm practices on nutrient availability and leaching to soils and streams. These are potentially correlated with fertilizer use and crop production, and may include additional nutrient inputs such as those from manure application and the effects of various farm management practices such as rotations, harvesting, and conservation tillage. For TN, fertilizer use was found to be a highly significant predictor of stream load in all six of the regional models. For the Northeast (MRB1), the model provided greater specificity by identifying separate contributions from fertilizer applied to the crop rotation group corn/soybean/alfalfa and to other crops (Moore *et al.*, this issue). Similar results were observed for TP in all cases except for the Southeast (MRB2) (García *et al.*, this issue) and Pacific Northwest (MRB7) (Wise and Johnson, this issue) models. In both cases, a more general measure of agricultural inputs based on agricultural land and combined inputs of fertilizer and manure (respectively) were found to be better predictors than fertilizer use alone.

Calibration results using manure generation estimates were similar to those based on estimates of fertilizer use in that they were statistically significant in most models, but more refined measures based on confined and unconfined livestock operations were identified in some. Nutrients associated with livestock waste reflect contributions from the excreted wastes of *unconfined* animals on farms, pastures, and rangelands and from the excreted wastes of *confined* animals, including those in concentrated animal feeding operations. Confined animal wastes include recoverable manure that may be applied to nearby farmlands as well as unrecoverable manure that is lost during the collection, storage, and treatment of the waste. In most of the models, estimates of the total amount of nutrient mass originating from manure were a significant predictor of stream nutrient loading, but separate estimates of confined and unconfined animal nutrient generation were statistically stronger predictors in some cases (MRBs 3 and 5). The best predictors for TN stream load included manure nutrient contributions from confined animals (MRBs 3 and 5) and unconfined animals (MRB5). For TP, separate estimates were also obtained for confined and unconfined animal operations in the Upper Mississippi (MRB3) model. In the Pacific Northwest (MRB7) model, agricultural predictors of nutrient load were relatively poorly resolved and only one variable, defined as the total mass of nutrients in fertilizer and manure, was found to be statistically significant (Wise and Johnson, this issue).

In all of the regional models, atmospheric deposition of nitrogen was found to be a statistically significant predictor of stream TN load, with the exception of the marginally significant contributions in the Missouri Drainage (MRB4) (*p*= 0.057) and Pacific Northwest (MRB7) (*p*= 0.105) models. In these two models, the coefficient estimates were lower than those in the other models, possibly due to lower runoff quantities in general and to the lack of major sources of atmospheric nitrogen in those regions. For all the other models, coefficients ranged from 0.22 to 0.51, and atmospheric nitrogen loadings to streams were larger than in the Missouri and Northwest regions. The higher coefficients in the models for the eastern, wetter regions indicate that a larger spatial change occurs in stream nitrogen load per unit change in wet-nitrogen deposition in these basins than in the western basins; this response may reflect the effect of the generally steeper gradients in precipitation, dry oxidized and reduced nitrogen deposition forms, and the nitrogen emissions from power plants and vehicles. By contrast, the lower coefficients in the models for the western, drier regions of the U.S. potentially reflect the generally flatter gradients in precipitation, dry deposition, and nitrogen emissions. Regional spatial patterns similar to these have been observed for wet and dry deposition of inorganic forms of nitrogen in the U.S. based on data collected as part of the CASTNET deposition monitoring conducted during the 1990s (e.g., Baumgardner *et al.*, 2002).

The atmospheric deposition estimates used as input to the regional SPARROW models are based on the use of wet inorganic nitrogen deposition measurements (nitrate plus ammonia) at National Atmospheric Deposition Program (NADP) sites as a proxy for total (wet plus dry) nitrogen deposition. Thus, SPARROW estimates of the nitrogen deposition delivered to streams (i.e., coefficients in Table 1) would be expected to account for additional contributions from dry nitrogen deposition forms because the regional patterns of wet and dry deposition are generally correlated over large areas of the U.S. (Baumgardner *et al.*, 2002; Holland *et al.*, 2005). On the basis of nitrogen deposition measurements from monitoring sites in the eastern and western U.S., dry deposition of inorganic nitrogen has been estimated to account for approximately 30% of the total dry plus wet inorganic nitrogen deposition (Baumgardner *et al.*, 2002). The SPARROW estimates of atmospheric nitrogen contributions to streams would also be expected to reflect regional atmospheric nitrogen sources, given that NADP wet-deposition nitrate estimates generally reflect regional patterns in NO_*x*_ emissions from stationary sources (Elliott *et al.*, 2007). However, in regions where the spatial distribution in wet atmospheric deposition of inorganic nitrogen is strongly influenced by inorganic ammonia deposition (e.g., MRB5, Lower Mississippi and Texas Gulf), the SPARROW estimates of atmospheric contributions to streams may primarily reflect agricultural sources of nitrogen (Rebich *et al*., this issue) that are largely associated with ammonia emissions from livestock (Holland *et al.*, 2005). Local atmospheric nitrogen sources, such as those attributed to vehicle emissions, are likely to be included in the SPARROW estimates of the nitrogen contributions from other modeled sources, especially urban sources (e.g., developed or impervious land).

In all regional models, diffuse anthropogenic and/or natural background sources of phosphorus were identified as significant contributors to stream TP loads. In three of the six models, forest land was identified as a statistically significant source of phosphorus to stream loads. In these cases, coefficient estimates ranged from 2.04 to 14.7 kg/km^2^/year, which correspond to stream exports that are appreciably less than those associated with anthropogenic land-based sources such as urban land (Table 2B); these estimates generally agree with reported exports of phosphorus from forested and urban catchments (Beaulac and Reckhow, 1982; NRC, 2009). Four of the six models include source variables defined by measures of stream bed phosphorus concentration or other channel characteristics. In these cases, the model simulation results imply that phosphorus originates from stream bed erosion and subsequent resuspension of phosphorus attached to sediment bed material. Stream bed sediments are known to accumulate phosphorus over long periods of time through sorption and settling processes and thus may reflect the legacy of phosphorus contributions from many upstream anthropogenic and natural sources, including agricultural areas, forests, and natural geologic sources. The phosphorus stored in bed sediments can be mobilized by storm flows and in that way contribute to stream TP loads (Sharpley *et al.*, 2008). Given the long-term annual time frame on which SPARROW models are based, settling/storage and resuspension processes are not separately quantified on a dynamic basis. Rather the SPARROW findings provide evidence that stream channels (and potentially their associated floodplains) are a prominent net source of phosphorus in some regions. Both the forest and stream sources may represent similar processes whereby soil or sediment bound phosphorus originating from natural or anthropogenic sources may be mobilized.

### Spatial Distribution and Magnitude of Identified Nutrient Sources

**FIGURE 2:**
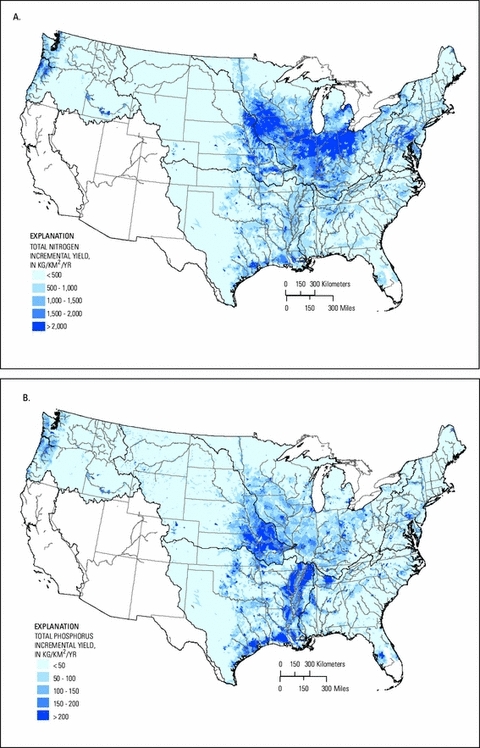
Spatial Distribution of Incremental Yields of (A) Total Nitrogen and (B) Total Phosphorus Simulated by SPARROW Models of Six Major River Basins.

The spatial distribution of TN and TP yield simulated using the six regional models for incremental stream-reach drainages is illustrated in Figure 2. Areas of high TN yield are apparent in the upper Midwest, where agricultural sources tend to dominate (Robertson and Saad, this issue), and intermittently throughout the country in a spatial pattern typically observed in areas downstream from urban and point sources. Areas of high TP yield are also predicted in the Midwest, but such areas are more extensive in the lower Missouri drainage and in the Mississippi delta region. The spatial pattern of TN and TP yield appear to be consistent across the six regions with no obvious discontinuities at regional boundaries. We interpret such consistency in the spatial pattern of prediction as an indication that the models provide a similar level of predictive capability for total TN and TP yield in all regions.

**FIGURE 3:**
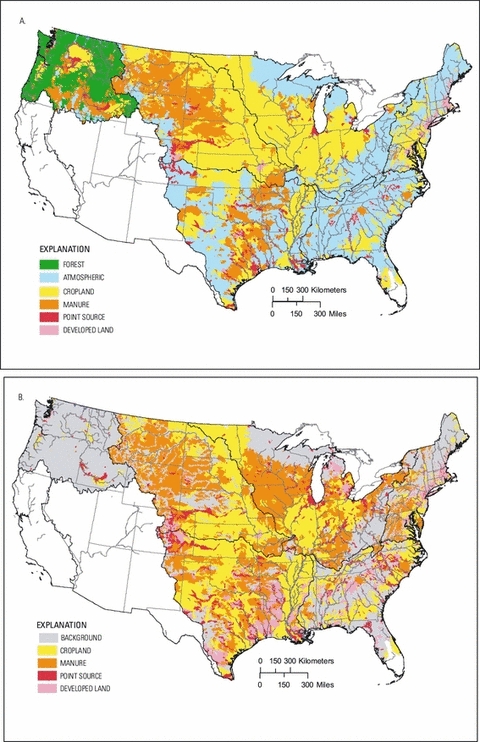
Spatial Distribution of the Largest Sources of (A) Total Nitrogen and (B) Total Phosphorus as Estimated Using Six Separate Regional SPARROW Models. Sources are aggregated in classes that are less detailed than those defined for the models individually and include point sources, developed land, crops (i.e., fertilizer application), manure generation, atmospheric deposition, and background sources.

As one evaluation of the consistency of nutrient sources identified in the regional models, we report (Figure 3) the major source for each stream reach, based on the largest percentage contribution of nutrient mass relative to the total stream load. Figure 3 illustrates the spatial distribution of sources identified in each region with the greatest contribution to TN or TP load in each stream reach. In some cases, however, the “major source” classification reflects only a marginally greater contribution of this source from among multiple sources that contribute nearly equally to total stream load. Thus, Figure 3 is primarily intended to provide an approximate yet regionally consistent synthesis of the locations of the largest contributing sources. The maps of major TN and TP sources reveal a number of important spatial patterns both across and within specific regions. Urban sources – point sources and developed land – are identified as the major local contributor of one or both nutrients in most regions. For example, such sources in the urban areas that extend from Washington, D.C. to Boston, Massachusetts are clearly identified as the major sources of both TN and TP. In urban areas such as Chicago, Illinois point sources are identified as the major source of TN and TP to local streams. Point sources affect water quality for long distances downstream in locations where these sources discharge to large rivers and instream attenuation is relatively small. This is best illustrated in Colorado, where point sources near the eastern slope of the Rockies continue to be the major source of nutrients in rivers such as the Platte, which drains far to the east.

The models indicate that both sewage discharge and urban runoff affect water quality over a large scale, but their effects occur in different ways, with urban runoff affecting more streams and sewage effluent contributing more mass overall (see Table S2 in the Supporting Information for more details). Of those streams in which urban runoff and sewage discharge are the largest sources of nitrogen (i.e., contributing more than 50% of the nitrogen mass to streams), diffuse urban runoff is the larger of the two sources of nitrogen in 43-98% of the stream reaches. Similarly for phosphorus, urban runoff is the larger of the two sources in 45-98% of the streams. This analysis considers the frequency with which the two types of urban sources (point and diffuse) predominantly affect streams. By contrast, comparisons on the basis of the total mass of nutrients delivered to streams by these two sources indicate that wastewater contributes from 73 to 85% of the total mass of nitrogen to urban streams and from 69 to 92% of the mass of phosphorus (Table S3).

Agriculture is a dominant source of nutrients throughout much of the center of the country (Figure 3). Commercial fertilizer and other sources of nutrients associated with cultivation (e.g., manure applied as fertilizer, N fixation by legumes, mineralization) are the major sources of nitrogen throughout the upper Midwest, whereas manure is the dominant nitrogen source throughout much of the upper Missouri, lower Mississippi, and western Gulf of Mexico drainages. Manure also is identified as the major source of phosphorus in many areas of the central U.S. as well as in parts of the Southeast and Mid-Atlantic regions. The separate contributions of nitrogen from confined and unconfined animal operations were statistically quantified in the Lower Mississippi (MRB5) region, whereas for phosphorus this separation was feasible only in the Upper Mississippi. In the other regions, manure nutrient generation is defined as the sum of contributions from both confined and unconfined animals except for the Upper Mississippi (MRB3) nitrogen model, where it is defined only as that from confined animals.

Overall, there is general consistency across the regions in the model predicted spatial patterns of major sources of nutrients. The importance of agriculture as the major source of nutrients extends across most of the Midwest, including contiguous areas of at least three of the model regions. Furthermore, the spatial distributions of crops and manure as the major sources of nutrients appear to be consistent across much of the country. Crops are identified as the major source of nitrogen in spatial patterns that extend in similar ways across contiguous parts of the Midwest (MRBs 3, 4, and 5). Manure is identified as the major source of nitrogen in the western and eastern portions of MRB4 and MRB5. Manure is identified as the major source of phosphorus on a much more widespread basis in all of the regions except MRB7, and the spatial patterns extend in similar ways across boundaries. Areas where manure is identified as the major source of phosphorus, for example, extend from north to south along the western portions of MRBs 4 and 5 and immediately west of the Mississippi across MRBs 3, 4, and 5.

Some inconsistencies are apparent in the identification of major sources of nutrients in watersheds along some of the regional boundaries (Figure 3). In some cases, these inconsistencies indicate important differences in the specifications and sensitivities of the models that demonstrate the challenges of fully accounting for local, subregional variability in processes within the area represented by a regional-scale model. For example, an abrupt difference in model predictions of the major sources of both nitrogen and phosphorus in the Pacific Northwest (MRB7) is apparent at the boundary of the model for the Pacific Northwest with the model for the Missouri Drainage (MRB4), which is defined by the continental (Rocky Mountain) divide (Figure 3). The differences in the model predictions are strongly influenced by the predominance of agriculture in the Missouri Drainage and of forests in the Pacific Northwest. Nutrient sources in the predominantly forested watersheds along the Rocky Mountain boundary are perhaps less accurately reflected in the MRB4 model, which lacks a specific source term for forests. Inconsistencies between the two models along the Rocky Mountain boundary may also be related to a general underrepresentation of nutrient loads in the models for these areas, which are generally difficult to estimate precisely due to the low density of stream monitoring sites and the relatively low magnitude of the loads. In contrast to these patterns, seemingly abrupt spatial differences in the “predominant source” classification (Figure 3) may also indicate cases where only marginal differences actually exist in the model predictions of source contributions. For example, different types of agricultural sources of TP (manure *vs.* crops) are identified as the predominant sources by the Upper Mississippi (MRB3) and Missouri Drainage (MRB4) models for watersheds along the boundary of the Upper Mississippi basin (Figure 3B), although the relative contributions to streams from these sources differ only by a small percentage.

In general, the importance of different types of agricultural sources varies somewhat by region. In regions of the eastern U.S. (MRBs 1 and 2), crops are larger sources of nutrients to agriculturally dominated streams than are animal wastes (see Table S2c). By contrast, animal waste is the dominant source of nutrients in some western regions such as the Missouri Drainage (MRB4), where it was the largest source of both nitrogen (51%) and phosphorus (63%) in agriculturally dominated streams. However, on a regional mass basis, crops as compared to animal manure are the largest source of nutrients in agriculturally dominated streams in nearly every region (47-79% for TN and 48-76% for TP as shown in Table S3c). In summary, the model results indicate that crops tend to be the dominant agricultural source of nutrients in most of the country, but animals tend to have a greater impact on stream nutrient loads in some parts of the western U.S.

**FIGURE 4:**
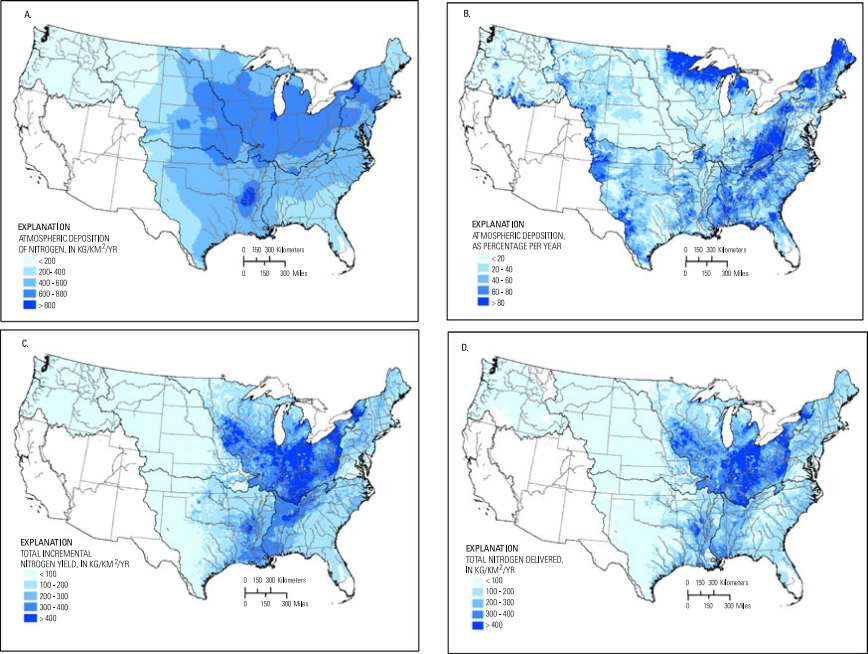
Spatial Distribution of Atmospheric Nitrogen: (A) From Deposition; (B) as Percentage of Total Input to Incremental Drainages; (C) as Yield From Incremental Drainages; and (D) as Yield Delivered to Downstream Drainages.

SPARROW model results provide multiple perspectives for understanding the spatial extent of the effects of individual sources of nutrients on water quality in streams and receiving waters. As part of our evaluations of the consistency of the nutrient sources in the regional models, we compared the model predictions of mass loadings for different model regions, including nutrient generation and delivery to streams and to downstream waters. Figure 4 illustrates the results of these comparisons for atmospheric nitrogen by showing the regional results for locations where nitrogen is deposited to the land surface (Figure 4A), what the contribution of nitrogen is locally to streams relative to other sources (Figure 4B), how much nitrogen mass is exported from local catchments by individual stream reaches (Figure 4C), and how much of that export reaches downstream waters (Figure 4D).

The spatial pattern of the atmospheric deposition of nitrogen across the U.S., which is illustrated in Figure 4, indicates that the greatest deposition occurs through the upper Midwest and northeastern parts of the country. Figure 4B illustrates the spatial pattern in the relative contribution of atmospheric deposition to the total stream load. The relative contribution is greatest where other sources are generally absent, such as in the northern extremes of the Midwest and Northeast and along much of the Appalachian range. Figures 4C and 4D illustrate the spatial pattern of stream nitrogen loads that originate from atmospheric deposition (4C) and the amount of those loads (4D) that are delivered to downstream waters (defined as the furthest downstream or terminal reach in each region). Comparison of Figures 4C and 4D illustrate the effect of attenuation with stream transport. Those areas drained by smaller streams and far from the terminal reach export proportionally much less nitrogen to downstream waters because the rates of natural attenuation processes such as denitrification are greatest in smaller streams, and longer travel times provide more time for attenuation to occur (Alexander *et al.*, 2000). Thus, some areas of high local export of atmospheric nitrogen (Figure 4C) provide lower export to receiving waters (Figure 4D). In general, the spatial pattern in stream TN loads originating from atmospheric deposition is similar to the pattern of deposition itself. Differences in the patterns are attributable to spatial variability in the importance of other sources and the effects of nitrogen removal processes during transport. In all of the model-derived Figures (4B-4D), the spatial pattern is generally consistent across regions and consistent with that of deposition itself. However, the MRB3 model does appear to predict atmospheric deposition yields that are somewhat higher than those of adjacent regions as illustrated at the boundaries of that region.

### Delivery of Nutrients to Streams

Table 3 summarizes the calibration results among the regional TN and TP models for landscape delivery variables that were found to be statistically significant. Delivery variables are categorized as those enhancing or reducing delivery and are simply listed for each model to facilitate model comparisons in a limited space. The reader is referred to the full citations (Table 1) for additional descriptions of the delivery variables in each model. Environmental characteristics represent important distinguishing factors among the regions, so that differences in the delivery variables identified as significant might be expected among the models; the data in Table 3 confirm the existence of such differences. Environmental characteristics include climate, soil, physiographic, geomorphic, and hydrologic variables. The specific set of delivery variables in each region is a reflection of specific conditions in that region as well as the characteristics of the water-quality monitoring records and catchments of the sites used for calibration (see Schwarz *et al.*, this issue).

Despite the general diversity in the delivery variables, some are identified as statistically significant across most regions. For both nitrogen and phosphorus, climate variables were found statistically to be among the most important factors in explaining the spatial variability in stream nutrient load. For example, mean annual precipitation was statistically important in enhancing nutrient delivery in five of the six TN models and four of the six TP models, thus implying that those areas with higher precipitation had greater long-term mean annual rates of delivery of nutrients to streams. Mean annual precipitation tends to be important in those regions with the highest precipitation gradient (MRBs 4, 5, and 7), those in which wet areas can be easily distinguished from dry areas. However, it was also found to be important in the Upper Mississippi (MRB3) and Southeast (MRB2) regions. Mean annual temperature is a significant TN delivery variable in three of the regions reflecting lower delivery possibly due to the greater denitrification-related loss associated with higher temperatures. Temperature was significant especially in those regions with the greatest temperature gradients (MRBs 1, 3, and 4), where areas with lower annual temperatures are easily distinguished from those with more moderate temperatures.

**FIGURE 5:**
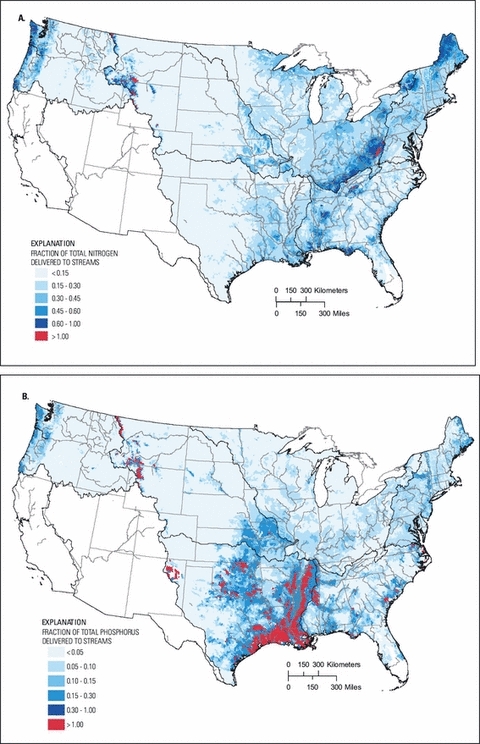
Fraction of (A) Total Nitrogen and (B) Total Phosphorus Delivered With Transport From the Land Surface to Stream Reaches.

Figure 5 illustrates the spatial pattern of the integrated effect of the climatic and landscape properties on delivery of nutrients to streams. We calculate the delivery fraction as the ratio of the amount of nutrient mass exported from each drainage divided by the amount input from sources in that drainage. The maps indicate that, in general, the spatial patterns of delivery are similar across the regional models. For example, mountainous areas and areas with high rainfall and low temperatures appear to have the greatest delivery rates of nitrogen to streams. Areas of high rainfall in the south central part of the country and the Pacific Northwest have the greatest delivery rates of phosphorus. Conversely, the lowest delivery rates of nitrogen occur in the central part of the country, where conditions are dry, and in the Southeast, where high temperatures may reduce delivery by increasing the potential for denitrification. Despite these general patterns, some model inconsistencies are apparent at certain regional boundaries. One example is TN delivery along the eastern boundary of the Upper Mississippi Drainage (MRB3), where the delivery rates are much higher than in either the Northeast (MRB1) or Southeast (MRB2). A second example is TP along the western boundary of the Missouri Drainage (MRB4), where delivery rates are much higher than they are in adjacent parts of the Pacific Northwest (MRB7). Such differences could be due to real environmental variations that affect delivery or to different emphases in the models caused by the distributions of calibration sites in each region.

### Loss of Nutrients in Streams and Reservoirs

The estimated long-term mean annual rate of nutrient removal in streams (Table 4) is reported as a first-order reaction rate constant. This expresses the nutrient removal as the fraction of the nutrient mass that is removed from the water column (via denitrification or long-term storage) per unit of mean water travel time in stream channels. The estimated rate constants are statistically significant for TN in all of the regional models, except for the Pacific Northwest (MRB7) model, and for four of the six TP models (exceptions include the models for MRBs 1 and 4). Estimates of the TN reaction rate constant ranged from 0.014 to 0.424 per day, which corresponds to an average removal percentage of from 1.4% to nearly 35% of the mean TN mass in streams per day of mean water travel time in channels. For TP, the comparable estimates for most regions range from about 5 to 26% removal of the TP mass per day of mean travel time, although the TP reaction rate constants for streams in the Southeast (MRB2) correspond to removal percentages that span a much larger range, from more than 50% in small streams to <1% in large streams. Note that the functional form of the Southeast model accounts for variations in the rate of stream loss associated with stream size through a continuous mathematical function as opposed to the discrete stream size classes used in the stream decay functions of the other regional models (García *et al*., this issue; Schwarz *et al.*, 2006).

**FIGURE 6:**
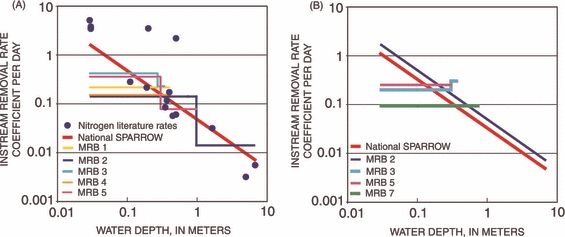
SPARROW Model Estimates of the Removal of Nutrients in Streams: (A) Total Nitrogen and (B) Total Phosphorus.

Total nitrogen removal rates estimated using the regional models indicate that the mean removal rates (i.e., reaction rate constants) decline with increases in mean water depth (related to stream size) in four of the major river basins (Figure 6A). This inverse relationship is consistent with that reported for previously developed national SPARROW TN models (Alexander *et al.*, 2000, 2008) and with current understanding of the hydrological and biogeochemical processes (denitrification, particulate settling, water velocity, and depth) that control nitrogen removal in natural waters and river drainage networks (e.g., Boyer *et al.*, 2006). This is illustrated in Figure 6A by the general agreement between the estimated TN reaction rate constants from the regional models and the rates of stream denitrification from the literature (Alexander *et al.*, 2008); the rates show similarities in both their magnitudes and their inverse relationship with water depth. The TN removal rate constants for the Southeast (MRB2) and the Lower Mississippi (MRB5) are statistically detectable across a wide range of water depths, from about 0.03 to nearly 10 m, whereas the removal rate constants reported for the other regions are statistically detectable only in streams with depths less than about 0.75 (MRB1) and 0.35 (MRB3, MRB4) m. TN removal rate constants were statistically identifiable for only one stream size class in the Missouri Drainage (MRB4) and no statistically significant TN removal is detected in streams of any size in the Pacific Northwest (MRB7). In general, the estimation of nutrient removal rate constants is expected to be more statistically challenging for larger streams because the rates of removal are generally expected to be small (Schwarz *et al.*, 2006; Alexander *et al.*, 2008).

The estimated instream TP removal rate constants show evidence of a decline in the rates with increases in water depth only for streams in the Southeast (MRB2; see Figure 6B). In streams of this region, the estimated TP removal percentages range from <5% per day of mean water travel time in streams with depths >1 m to more than 50% in small streams with depths <0.1 m. These estimates are about a factor of 1.5 higher than those estimated in a previous national TP SPARROW model (Alexander *et al.*, 2008), although both models display a similar inverse relationship with stream size (Figure 6B). In the other regions, the estimated mean TP removal percentages were reported to be less than about 30% per day of water travel time in small streams (i.e., those <0.75 m deep in MRB7 and <0.35 m deep in MRBs 3 and 5); these estimates are generally similar to or somewhat less than the estimates for the streams with identical water depths in the Southeast (MRB2). No statistically significant TP removal was detected in larger streams in the other regions (MRBs 3, 5, and 7; i.e., streams with mean water depths >0.75 and 0.35 m, respectively). No statistically significant TP removal was detectable for any stream size in MRBs 1 and 4, and in fact, larger streams were found to be a source of TP in MRBs 4 and 5, possibly due to TP associated with sediment and net erosion in those regions (Brown *et al*., this issue; Rebich *et al*., this issue).

The estimated long-term mean annual rate of nutrient removal in reservoirs (Table 5) is reported as a first-order mass transfer rate constant that is estimated in the model as a function of the mean annual areal hydraulic load (quotient of mean flow and reservoir surface area) in reservoirs. The first-order rate constant (units of length per time) describes the height of the water column (meters) from which nitrogen or phosphorus is removed per unit of time (year). Statistically significant removal rate constants are estimated for four of the six regional TN models and for five of the six TP models (Table 5). For TN, the removal rates are generally similar in MRBs 2, 4, and 5, ranging over a narrow interval of from 10.7 to 12 m/year. The TN removal rate for the Upper Mississippi (MRB3) is about one-half of these rates (6.7 m/year). All of the estimated TN removal rate constants are higher by a factor of 5-10 than that estimated in the previous national SPARROW TN model (Alexander *et al.*, 2008). For TP, the removal rate constants are among the largest in MRBs 2 and 4 (30 and 39 m/year, respectively), which bracket the rate constants estimated in the previous national SPARROW TP model (Alexander *et al.*, 2008). The TP removal rate constants estimated for reservoirs in MRBs 1, 3, and 5 (about 3-9 m/year) range from 10 to 25% of the removal rate constants estimated for reservoirs in MRBs 2 and 4.

Overall, the regional model estimates of the first-order removal rate constants for TN (i.e., mass transfer rate constants) are typically smaller for reservoirs than for streams by about 10-25%. This comparison of rate constants in the same region is based on the mass transfer rate constants for reservoirs (Table 5) and the mass transfer rate constants for streams that correspond to a reexpression of the reaction rate constants reported in Table 4 (calculated as the product of the reaction rate constant and the mean water depth). This pattern of nitrogen removal is generally consistent with the relative ordering of the first-order removal rate constants reported in the literature for stream and reservoir denitrification and with the ordering of the removal rate constants observed for these aquatic environments in the national SPARROW TN model (Alexander *et al.*, 2008). The lower supply of organic matter in reservoirs has been cited as a possible biochemical explanation for the smaller nitrogen removal rate constants in reservoirs compared to those for streams (Howarth *et al.*, 1996).

For phosphorus, larger removal rate constants are estimated in the models for reservoirs than for streams in MRBs 1, 2, and 4; a result that is generally consistent with the relative ordering of the removal rate constants estimated in the national TP SPARROW model (Alexander *et al.*, 2008). By contrast, similar comparisons for MRBs 3 and 5 indicate that the removal rate constants for small streams are approximately the same as those estimated for reservoirs, whereas the removal rate constants for large streams are generally larger by a factor of two to six than those for reservoirs. Regional differences between the stream and reservoir removal rates of TP suggest that different hydrologic and biogeochemical factors may be dominant in the aquatic ecosystems of these regions, including differences in the long-term effects of particulate burial, floodplain storage, and the speciation of phosphorus among particulate and dissolved phases.

## Discussion

The SPARROW model results provide a variety of types of information that can inform efficient design and implementation of water-quality management efforts. The statistical nature of the calibration process helps to identify which nutrient sources and which delivery factors are most strongly associated with long-term mean annual stream loads. The mass-balance framework of the models provides information to describe the relative importance of different contaminant sources and delivery factors. The spatial referencing of SPARROW models provides information about where each of those sources and delivery factors are most important. And lastly, the networking and instream processing aspects of SPARROW provide the capability of relating downstream loads to the appropriate upstream sources so that nutrient contributions from a variety of distant upstream sources can be systematically and accurately evaluated in relation to those loads. The regional models that are the subject of this paper provide these capabilities from a regional perspective, and comparison of the regional results provides a national perspective.

The regional models identify similar sets of nutrient sources as being important contributors to stream loads, but in some cases provide refined or more resolved definitions of these sources. For example, in all of the models urban and agricultural sources were identified as being among the most important contributors to stream nutrient loads and to downstream receiving waters. This is consistent with the findings of many watershed studies (NRC, 2000; Carpenter *et al.*, 1998) as well as of USEPA (2007) and state assessments of the major sources of nutrients in U.S. water bodies. In contrast to these prior studies, however, the SPARROW regional models provide a comprehensive assessment of the spatial extent of the effects of urban and agricultural sources on nutrient loads in relation to other sources both among and within major U.S. river basins. And in many cases the SPARROW models differentiate among specific types of urban and agricultural sources and provide an indication of their relative importance to stream nutrient loads.

For example, nearly all of the regional models were able to distinguish the importance of the nutrient contributions from urban point sources (municipal and industrial wastewater effluent) separately from those originating from diffuse urban sources. Diffuse sources were represented by surrogate measures of their contributions, including the area of developed land or of impervious surfaces (streets, parking lots, and roofs). This distinction between point and diffuse sources has important management implications for urban as well as nonurban watersheds in view of the difficultly of directly measuring and controlling diffuse urban sources. Diffuse sources may include direct runoff from impervious surfaces and inflows of groundwater whose chemical characteristics have been affected by such sources as fertilizers, septic systems, and atmospheric deposition from vehicle emissions. Furthermore, whereas urban sources are commonly the most important local source of nutrients to streams, less is known about the spatial extent of their downstream transport and their contributions to the nutrient budgets of receiving waters, especially those with large drainage areas of mixed land use. Results of simulations made with the regional models significantly advance the understanding of these urban sources in major U.S. river basins.

A key finding from the simulation results of the regional SPARROW models is that diffuse urban sources of nutrients can affect water quality in urban streams across broad areas, but that wastewater discharges contribute such large masses of nutrients to streams that they are often more important from the perspective of the mass balance of nutrients for an entire region. Thus, wastewater affects fewer streams but contributes a larger mass of nutrients that may be more important for the water quality of downstream receiving waters, but diffuse urban sources have a broader spatial impact by affecting a larger number of streams. Both perspectives are important and each may have specific relevance for different management objectives.

Agricultural variables were strongly associated with high TN and TP loading of streams in all six regions, but some regional differences were apparent in the types of agricultural production (i.e., cultivated crops *vs.* livestock) that affect water quality most broadly. In regions of the eastern U.S. (MRBs 1 and 2), crops were larger sources of nutrients to agriculturally dominated streams than were animal wastes (see Table S2c). More broadly, the model results indicate that cultivation is the dominant agricultural nutrient source in most of the country, but animals have a greater impact on stream nutrient loads in some areas of the western U.S. For these reasons, greater efficiency in national policy might be achieved by addressing regional differences in agricultural practices and by making implementation decisions at regional or local scales where they can be adapted to local practices.

Atmospheric deposition is a statistically significant source of nitrogen in all six regions for which models were developed. The Missouri drainage (MRB4) and Pacific Northwest (MRB7) were the only regions where it was marginally significant, presumably because of the absence of large, widespread sources of atmospheric nitrogen throughout most of these regions. Nitrogen deposition is greatest throughout most of the eastern half of the country, is the largest contributor in 50-79% of eastern streams (Table S2) and contributes 18-46% of the nitrogen mass in eastern catchments (Table S3). Atmospheric deposition is usually a small contributor of nutrients compared to agricultural, urban area, and point sources, but where those sources are not present deposition often represents the largest source of nitrogen to streams. Such areas include parts of northern Minnesota and Wisconsin, much of the Appalachian ridge, and the northern parts of New England. In such areas, eutrophication may be a minor concern or issue, but damage to ecological integrity and specifically to forest health from nitrogen deposition has been documented (Aber *et al.*, 2003). Thus, control of atmospheric sources of nitrogen is important for the protection of the ecological integrity of some otherwise un-impacted areas of the country.

In the development of most of the regional SPARROW models, it was found that accounting for highly diffuse anthropogenic or natural background sources of nutrients was important, but the sources that were most strongly associated with stream nutrient loads varied by region. For nitrogen, identification of background sources is complicated by the relative ubiquity of atmospheric deposition. In the eastern part of the country where atmospheric deposition sources are relatively high, it is likely that background sources (such as from forest areas) are accounted for in the mass balance as a minor part of the atmospheric deposition contribution. In the West, however, where atmospheric deposition of nitrogen is relatively low, background sources may be more important and thus statistically identifiable as a part of the overall mass balance. This was the case in the Pacific Northwest (MRB7) where both forest and atmospheric deposition were identified as important parts of the nitrogen mass balance (Wise and Johnson, this issue). For all of the regional TP models, background sources that contributed small quantities of phosphorus to the budgets of most streams in the regions were related to forested areas or stream bed erosion. In addition, the mining of geologic deposits of phosphorus was identified as a locally important source in the Southeast (MRB2) where it contributed the majority of the phosphorus loads in some streams. Overall background sources of phosphorus affect streams broadly and are the major contributors in 18-90% of streams (Table S2). But they contribute a relatively small mass locally in streams that, in some cases, made up a significant part of the regional nutrient budget; for example, background sources make up 6-37% of the total catchment yield in most regions and 60% in the Pacific Northwest, where forests dominate (Table S3). While highly diffuse anthropogenic or natural background sources of nutrients may not be manageable, it is important to account for them in setting appropriate water-quality goals. This may best be done regionally or locally, given the differences in environmental conditions among regions. The one exception would be atmospheric deposition of nitrogen, for which extraregional sources may affect stream TN loads in any given region.

The greatest regional differences among the models were observed in the environmental characteristics that affect nutrient delivery, including soils, physiographic, geomorphic and hydrologic variables that can enhance and attenuate delivery of nutrients to streams. Understanding the effects of these characteristics on nutrient delivery could be valuable for the development of more effective regional nutrient management plans. While in most cases the characteristics themselves cannot be changed or managed, knowledge of the magnitude of the nutrient load response to these factors is critical for prioritizing areas for management actions. Climatic variables were identified in most of the models as being important to the delivery of nutrients to streams, with areas of high precipitation associated with greater delivery of both nitrogen and phosphorus to streams. This would be expected given that watershed hydrology is the primary control of nutrient flux. Areas of low temperature were associated with higher delivery of nitrogen to streams, presumably due to lower rates of biologically mediated denitrification. These climate-related controls on nutrient loads are important environmental factors to consider when prioritizing management actions, especially in regions where the hydrologic and temperature gradients are steep (e.g., MRBs 1 and 4). However, the climatic variables are only one set of identified nutrient delivery factors and they emphasize the need to consider any environmental characteristics that affect nutrient delivery specifically in a given region.

In comparing the regional models, our results indicate that many aspects of the models are consistent nationally, but that regional differences exist due to variations in underlying geographic characteristics or to differences in the models’ abilities to fully represent all of the underlying processes. In nearly all cases, the regional models identified similar major categories of nutrient sources, including point sources, urban runoff, agricultural practices, and atmospheric deposition. However, identification of more detailed aspects of these major source categories, such as the type of agricultural practice or background source influencing stream nutrient loads, did differ among the regional models. Similarly, the regional models identified some of the same environmental characteristics affecting the delivery of nutrients to streams, but many differed by region. These differences in model results can be due to actual variations in regional geographic characteristics, and regional model specificity is important in these cases. However, the relationships between nutrient loads and some regionally important variables can be complex and poorly characterized by available data. Thus, to improve our ability to identify regionally important characteristics that affect stream nutrient loading, it is also important to improve the datasets that form the basis of modeling assessments.

The comparisons of the results of simulations made with the regional SPARROW nutrient models described in this paper provide insights into existing scientific questions regarding the nature of the effects of spatial scale and landscape heterogeneities on nutrient transport and how well watershed models can describe these processes. Overall, we found empirical evidence of the general universality (i.e., scale independence) in the effects of landscape and aquatic processes on water quality over broad spatial scales (though limited to the spatial dimensions associated with the river network datasets applied in the regional studies). This is demonstrated by similarities in the types of explanatory variables that were found to be statistically significant in the models (e.g., agriculture, urban, atmospheric deposition, precipitation, temperature) and the estimated values of the model coefficients that quantify the magnitude of the stream nutrient load response to these factors. However, important regional variations in these effects were also identified in simulation results from some of the models (e.g., the relative importance of forest land in the Pacific Northwest), including examples where local subregional effects were poorly quantified in the models (e.g., Rocky Mountain border of MRB4 and MRB7). The identification of regional and subregional differences in the models indicates the importance of latent processes for which proxy landscape variables are currently lacking or poorly described by the available geospatial data.

Determining whether regional differences exist in model-based descriptions of hydrological processes (functional forms and/or parameter values) is an intrinsically ambiguous matter, which is complicated by the absence of definitive guidance from prior literature studies, especially over regional and continental scales. As we note in the introduction, our study is meant to provide an initial step in informing ongoing related scientific discussions and investigations. Our study benefited from consistency in the general model structure and variable selection process employed by the regional modelers. Nevertheless, our comparison is highly descriptive and we do not view it as being definitive as to the causes of the regional differences, given that it is based on a postcalibration evaluation of independent regional models and given the likely importance of latent processes for which even proxy data for use as predictors in the models are lacking. Additional investigations will be necessary with SPARROW and other watershed models to better understand the appropriate regionalization and spatial scales required to accurately account for environmental processes and to determine whether sufficient geospatial data are available to serve as proxies for their effects on stream water quality. Additional evaluations of regional coefficients using a “fixed-effects” modeling approach (e.g., Schwarz *et al*., this issue) as currently used in SPARROW is, however, potentially limited by the challenge of identifying *a priori* the appropriate regional domains and spatial scale required for model parameters to accurately account for the effects of processes that affect nutrient loading and local and downstream water quality. Other approaches, such as Bayesian modeling, which assume model parameters to be random rather than “fixed” variables (e.g., Qian *et al.*, 2005), need to also be explored as these may potentially provide important insights into the nature of these latent processes.

All of the regional SPARROW models were developed with similar prediction accuracy, as defined by statistical diagnostics (i.e., RMSE, *R*^2^). However, many of the datasets used to develop the models are limited in specific ways, and model uncertainty is likely to be attributable in part to those limitations. Model prediction accuracy did appear to be related to the number of calibration sites available in each region as well as the certainty in the load estimates from those sites. This result indicates the value of well-established, long-term monitoring programs that are adequate for capturing the variability of contaminant loading. Geospatial data that describe nutrient sources are also critically important to model calibration, but such data are commonly collected for other objectives and need to be adapted for use in modeling. Two key examples are the datasets available for documenting point and agricultural sources. In both cases, the datasets required for model input must be developed by extending or extrapolating information collected for other objectives, thus adding uncertainty to any modeling analysis. Use of models to address water-quality management and resource allocation objectives need to take such uncertainty into account. National coordination of the development of monitoring and geospatial databases will provide benefits for water-quality assessment across national, regional, and smaller spatial scales.

## Summary and Conclusions

Comparison of simulation results from 12 independently calibrated SPARROW models of stream nutrient loads in major river basins across the U.S. reveals some national consistencies and some regional specificity of nutrient sources and delivery factors. All of the models provided similar levels of prediction accuracy as defined by statistical diagnostics (i.e., RMSE, *R*^2^). The models were generally consistent in identifying major sources although refined measures of those sources were better predictors of stream nutrient loads in some regions. Urban and agricultural sources of nutrients were the largest contributors to stream loads in most of the regions. The more diffuse of these sources (e.g., urban runoff) tended to affect water quality in more streams, whereas the more focused sources (e.g., point sources) commonly contributed the largest mass of nutrients regionally, which implies that they could have greater effects than do diffuse sources on downstream water quality. Agricultural sources were consistently identified as significant sources of nutrients throughout the country, but the relative importance of crops and animal waste to stream nutrient loads varied somewhat by region. Climatic factors were identified by most of the models as being important regional delivery factors for nutrients, but many other delivery factors appeared to be regionally specific. Spatial patterns in the source contributions and delivery rates were generally consistent, but some differences were apparent at regional boundaries. Finally, in all cases the accuracy and precision of the models are limited by the data available to build them. Thus, there is an ongoing need to expand monitoring efforts in order to better identify the factors that control stream nutrient loading and to improve the resolution of geospatial datasets to provide better explanatory power for all models used to support water-quality management decisions.

## SUPPORTING INFORMATION

Additional Supporting Information may be found in the online version of this article. The reader is supplied with information on integrated analysis of model residuals and the identification of major sources of nutrients.

Please note: Neither AWRA nor Wiley-Blackwell is responsible for the content or functionality of any supporting materials supplied by the authors. Any queries (other than missing material) should be directed to the corresponding author for the article.
