# Supplementary material for: Factors Affecting Stream Nutrient Loads: A Synthesis of Regional SPARROW Model Results for the Continental United States
Source: J Am Water Resour Assoc. 2011 Oct;47(5):891–915. doi: 10.1111/j.1752-1688.2011.00577.x (PMC3307615; doi:10.1111/j.1752-1688.2011.00577.x)
Supplement: Supplementary file 1 [file jawr0047-0891-SD1.doc]

Factors Affecting Stream Nutrient Loads: A Synthesis of Regional SPARROW Model Results for the Continental United States

*Stephen D. Preston, Richard B. Alexander, Gregory E. Schwarz and Charles G. Crawford*

# Supporting Information

Integrated Analysis of Model Residuals

To systematically investigate the spatial differences in the prediction errors of the regional models, we perform a regression analysis to relate SPARROW model residuals from all of the regional models to specific attributes of the calibration sites. The dependent variables in these regressions are the squared natural logarithm of the residual error terms of the SPARROW models. The independent variables include an intercept, size of the drainage basin at the calibration site, a measure of the coefficient of variation (COV) of the mean load estimate used in the SPARROW model calibration and binary variables to account for differences in the mean residual for each region. Drainage area is included to detect potential effects of spatial scale on SPARROW model residual error. Load estimate COV was included to detect potential effects of load estimation error on SPARROW model residual error. And the regional binary variables were included to detect significant differences in the amount of residual error among the six regional models for total nitrogen (TN) and total phosphorus (TP).

Significant relations between SPARROW model error and the site attributes are indicated by the results of the regression analysis (Table S1). Both the TN and TP regressions were highly significant as indicated by F statistics, although the coefficients of determination (R^2^) were low implying that the regressions accounted for only a small amount (8-11%) of the variation in model residuals. Low R^2^ values might be expected for

this type of analysis since most of the variation in the data has already been accounted for by the SPARROW models themselves. The regression analysis described in this section is designed to evaluate any remaining structure in the combined set of residuals from all of the six regions. For both the TN and TP regressions, the coefficient associated with drainage area was significant and negative implying that SPARROW model residual error increases with decreasing basin size. Thus the model errors tend to decline with progression downstream and as smaller basins are aggregated. For both TN and TP models, uncertainty in the load estimate used for calibration was statistically significant and positive predictor of the magnitude of SPARROW model residual error implying that SPARROW model error increases with greater uncertainty in the load estimate.

In most cases the “regional effect” variables in the regression models are statistically significant (α = 0.05) predictors of the magnitude of SPARROW model residual error among the six regional TN and TP models indicating that the amount of error in the SPARROW models varies by region. The regional effect coefficients were not statistically significant in three cases including the TN model in MRB5 and the TP models in MRB5 and MRB7. This implies that the residual error in these two cases is not statistically different than the mean of all models. To provide an indication of the relative magnitude of error in each region, mean regional accuracy estimates are provided in Table S1 and are approximately equal to the standard error of an arbitrary reach's predicted load in a given region, expressed as a percentage of the prediction. Estimates of the regional accuracy differential, defined as the difference between the accuracy of a specific region and the average regional accuracy, are provided as an additional indication of the relative error among the regional models.

The magnitudes of the coefficients and of the accuracy estimates indicate that there is an east / west pattern in the amount of residual error of the models. Regions 1, 2 and 3 have negative regression coefficients and negative accuracy differentials in all cases implying that the magnitude of SPARROW model residual error is lower than average in these regions. Accuracies in regions 1, 2 and 3 range from 21 to 39 percent for the TN models and from 46 to 54 percent for the TP models. By contrast regions 4, 5 and 7 have positive coefficients and positive accuracy differentials implying that model residual error is higher in these regions. Accuracies in regions 4, 5 and 7 range from 47 to 69 percent for the TN models and from 67 to 96 percent for the TP models. Region 4, the Missouri basin, has the highest model residual variance of all regions and is particularly large for the TP model which has an accuracy differential that is 30 percent greater than the mean.

One potential reason for these differences is that processes related to nutrient transport in the arid west may not be as fully characterized by the models. In addition, monitoring in the western states tends to be more sparse (see Table 1 in the body of the paper) where conditions are more arid and population numbers are lower. The combination of limited characterization of transport processes in the west and lower numbers of calibration sites may be the cause of higher SPARROW model residuals for the western regions. However, the spatial consistency of these findings for both TN and TP suggests that the true unobserved processes that cause these differences are likely to be the same for both nutrient species.

Identification of Major Sources of Nutrients

To better understand the effect of sources of nutrients on overall stream loads, we compiled statistics to evaluate the relative importance of each major type of source on a regional basis. For both TN and TP models, we compiled estimates of the percentages of stream reaches for which each source was the largest contributor (Table S2). We then subdivided these percentages further for two of the major source categories including urban and agricultural sources. For urban sources, we identified those stream reaches for which the combination of urban runoff and sewage treatment plant discharge was the largest source and then calculated the percentage of those streams for which each of those two

sources was the largest (Table S2(b)). Similarly, for agricultural sources, we identified those stream reaches for which the combination of crop fertilizer and animal waste was the largest source and then calculated the percentage of those streams for which each of those two sources was the largest (Table S2(c)). The above estimates represent the frequencies with which each source impacts stream reaches as defined for each model. For an alternative perspective, we performed similar analyses on a mass basis for each region. In this second set of analyses, we summed the nutrient flux originating from stream reach drainages overall and by source, and then calculated the percentages of the total regional flux contributed by each source (Table S3).

The two assessments of sources described above provide different perspectives on the roles of the major sources of nutrients in defining stream fluxes in each region. Those perspectives provide complementary information and each may have relevance for managing nutrient loads. Table S2(a) indicates that urban sources such as urban runoff and sewage treatment plants are not the largest sources of nutrients for most streams. Diffuse sources such as agricultural inputs, atmospheric deposition of nitrogen and background sources of phosphorus are the largest sources of nutrients for most streams throughout the six regions evaluated. In many cases, however, the largest sources can be quite small for drainages where nutrient loads are low. Thus it is important to also evaluate sources of nutrients on a mass basis in addition to a stream reach frequency basis. Table S3(a) indicates that on a mass basis urban sources play a much greater role in defining the amount of nutrients reaching streams on a regional basis. Based on this analysis urban sources, including urban runoff and sewage treatment plants, contribute from 12 to 48 percent of the nitrogen in stream loads and from 19 to 63 percent of the phosphorus depending on the region considered.

A similar result is found when considering only those streams that are affected most by urban sources. Table S2(b) indicates that in most regions ( i.e. – regions 1, 4 and 7), the more diffuse urban runoff is the largest contributor of nitrogen to urban streams and in 2 other regions (i.e. – regions 2 and 5) urban runoff is the largest contributor in 43-44 percent of urban streams. Those numbers are even higher for phosphorus for which urban runoff is the largest contributor in 45 to 98 % of urban streams. Those results appear to be different when evaluating sources of nutrients to urban streams on a mass basis. Table S3(b) indicates that sewage treatment plants contribute the largest mass of both nitrogen (73 – 85 percent) and phosphorus (69-92 percent) to urban streams in all six regions. Each of these results is important and complementary, but their relevance may depend on the management goals being addressed. If the management goal is to protect the largest number of streams, then urban runoff might receive higher priority for management since it is the largest contributor to most urban streams. Sewage treatment plants affect fewer streams but contribute much larger quantities of nutrients and thus their management may be more important if limiting the integrated effect of urban sources is most important as it might be for protecting the quality of receiving water bodies.

Evaluation of agricultural sources does not provide as clear of a differentiation as that for urban sources (Tables S2(c) and S3(c)). Crop fertilizer and animal waste sources were compared and crop fertilizer sources tends to be the largest contributor of both nitrogen and phosphorus on both a stream reach frequency basis and on an overall mass basis. On a stream frequency basis, crops are the largest contributor of nitrogen in from 32 to 95 percent of agriculturally dominated streams and the largest contributor of phosphorus in from 37 to 87 percent of agriculturally dominated streams. On a regional mass basis, crops are the largest contributor of nitrogen in from 47 to 79 percent of agriculturally dominated streams and the largest contributor of phosphorus in from 48 to 76 percent of agriculturally dominated streams. For nitrogen only in the Pacific Northwest (MRB7) do animals appear to clearly play a larger role than crops in most agriculturally dominated streams and to a lesser degree on a regional mass basis. For phosphorus only in the Upper Midwest (MRB3) and the Missouri River basin (MRB4) do animals appear to play a larger role than crops in most agriculturally dominated streams and to a lesser degree on a regional mass basis. These results are different than those of urban sources most likely because both types of agricultural sources tend to be diffuse in nature. The degree to which they are diffuse may vary by region and thus both sources appear to play a similar role in the number of streams they affect and in the overall mass contribution (i.e. – regions 4 and 5).
